# Trends and factors related to adolescent pregnancies: an incidence trend and conditional inference trees analysis of northern Nicaragua demographic surveillance data

**DOI:** 10.1186/s12884-021-04215-4

**Published:** 2021-11-05

**Authors:** Wilton Pérez, Katarina Ekholm Selling, Elmer Zelaya Blandón, Rodolfo Peña, Mariela Contreras, Lars-Åke Persson, Oleg Sysoev, Carina Källestål

**Affiliations:** 1 Department of Women’s and Children’s Health, Uppsala University, Uppsala, Sweden; 2 Institute of Nutrition of Central America and Panama (INCAP), Calzada Roosevelt 6-25, Zona 11, Guatemala, Guatemala; 3 Asociación para el Desarrollo Económico y Sostenible de El Espino (APRODESE), Chinandega, Nicaragua; 4 Nicaraguan Autonomous National University, León (UNAN-León), León, Nicaragua; 5 Pan American Health Organization, Tegucigalpa, Honduras; 6 Department of Disease Control, London School of Hygiene & Tropical Medicine, London, UK; 7 Department of Computer and Information Science, Linköping University, Linköping, Sweden; 8 Department of Dental Research, Public Dental Service, Region Örebro County, Faculty of Medicine and Health, Örebro University, Örebro, Sweden

**Keywords:** Adolescent pregnancies, Incidence trend, Adolescent birth rate, Adolescent pregnancy rate, Conditional inference trees, Data mining, Predictors

## Abstract

**Background:**

We aimed to identify the 2001–2013 incidence trend, and characteristics associated with adolescent pregnancies reported by 20–24-year-old women.

**Methods:**

A retrospective analysis of the Cuatro Santos Northern Nicaragua Health and Demographic Surveillance 2004–2014 data on women aged 15–19 and 20–24. To calculate adolescent birth and pregnancy rates, we used the first live birth at ages 10–14 and 15–19 years reported by women aged 15–19 and 20–24 years, respectively, along with estimates of annual incidence rates reported by women aged 20–24 years. We conducted conditional inference tree analyses using 52 variables to identify characteristics associated with adolescent pregnancies.

**Results:**

The number of first live births reported by women aged 20–24 years was 361 during the study period. Adolescent pregnancies and live births decreased from 2004 to 2009 and thereafter increased up to 2014. The adolescent pregnancy incidence (persons-years) trend dropped from 2001 (75.1 per 1000) to 2007 (27.2 per 1000), followed by a steep upward trend from 2007 to 2008 (19.1 per 1000) that increased in 2013 (26.5 per 1000). Associated factors with adolescent pregnancy were living in low-education households, where most adults in the household were working, and high proportion of adolescent pregnancies in the local community. Wealth was not linked to teenage pregnancies.

**Conclusions:**

Interventions to prevent adolescent pregnancy are imperative and must bear into account the context that influences the culture of early motherhood and lead to socioeconomic and health gains in resource-poor settings.

**Supplementary Information:**

The online version contains supplementary material available at 10.1186/s12884-021-04215-4.

## Background

Adolescent pregnancies may have adverse consequences for the mother as well as the baby. Teenage mothers may have to interrupt or limit their education with consequences for employment and future income [1]. They run a higher risk of obstetric complications, including preeclampsia, fetal growth restriction, and preterm birth due to biological immaturity [2, 3]. The children may be disadvantaged at birth with increased risk for low birth weight and stunted linear growth. These children more often fail to complete secondary school [4]. In low- and middle- income countries, complications of adolescent pregnancy and childbirth are leading causes of death in this age group [5].

The 2030 Agenda for Sustainable Development Goals (SDGs) [6] and the United Nations Global Strategy for Women’s, Children’s, and Adolescents’ Health [7] identified adolescent pregnancies as an appropriate indicator. The agreed SDG indicator is the adolescent birth rate (ABR), which is the number of births per 1000 women 10–14 and 15–19 years of age, respectively. Besides the ABR, adolescent pregnancy rates (APR) are also reported, including ongoing pregnancies, abortions, and stillbirths per 1000 women 15–19 years of age. Commonly these indicators are calculated using data retrospectively reported by 15–19-year-old women. In their 2013 report, the United Nations Population Fund stated that retrospective data from 20 to 24-year-old women provide better estimates, as reports from 15 to 19-year-old women censor data from the younger women who still face the risk of pregnancy before they reach the age of 19 years [8].

The WHO statistics from 2018 [9] indicate that there are annually 12.8 million births to mothers aged 15–19 years, corresponding to 44 births per 1000 women in that age group. Globally, ABR varies with the highest rates in Sub-Saharan Africa and the lowest in Western Europe and Central Asia. The global median ABR has, as reported in 2012, declined by 40% since the 1990s [10]. Latin America and the Caribbean have, however, experienced the slowest decline of all regions in the world [11, 12]. This lower decrease in ABR is notable since this region has had a substantial decline in overall fertility [13]. Central America has a majority of this region’s high-ABR countries [11].

In a study with an ecologic design including 162 countries, adolescent pregnancies were negatively associated with national wealth (per capita gross domestic product or GDP) and expenditure on education as a percentage of GDP and positively linked to income inequality (Gini index) [10]. A systematic review focusing on low- and middle-income countries [14] reported associations between adolescent pregnancies, low educational levels, and insufficient access to contraception. Teenage pregnancies more regularly occur in settings where early marriage and early sexual debut are common, more frequently occurring in rural areas and among ethnic minority groups [14]. Educational level and household wealth have consistently been associated with adolescent pregnancies [11, 15, 16]. A systematic review focusing on adolescent pregnancies in Sub-Saharan Africa pointed at the importance of community and national contextual factors in addition to individual or household level factors behind adolescent pregnancies [17].

A technical consultation on adolescent pregnancies in Latin America [11] stressed that multi-layered factors contribute to the occurrence and distribution of early pregnancy. Such factors were limited information on sexual and reproductive health, restricted access to sexual and reproductive health services including effective contraception, sexual violence, and unfavorable gender norms. Importantly, the status of motherhood might be a pathway out of poverty that can lead to early marriage and greater acceptance of early pregnancies. For some, pregnancy may be unintended and unwanted, while for others, it implies adult status and upward social mobility [18].

Recently (2018), the Council of Ministers of Health of Central America and the Dominican Republic (COMISCA) approved the regional strategic plan for preventing pregnancy in adolescence for each country to contextually adapt and implement [19]. The plan called for strengthening of the health and educational systems, adolescent empowerment, policies against violence, health promotion, and evidence generation. Despite this, there is an urgent need of recent scientific assessment of adolescent pregnancies and related determinants in Central America.

Nicaragua has consistently reported high adolescent birth and pregnancy rates, although with a slow decline [11, 13]. The 2019 PAHO report stated that ABR for 15–19-year-olds was 83.3 per 1000 women [20]. The Northern Nicaragua Health and Demographic Surveillance System (NN-HDSS) includes demographic and reproductive data as well as household and individual characteristics. The NN-HDSS may target either a whole population area or a representative sampling frame. The NN-HDSS starts with a population and household baseline census followed by regular updating rounds to collect vital event information (i.e., births, deaths, immigration, and outmigration) and health-relevant outcomes. By 2021, the number of HDSS [similar to our NN-HDSS] registered in the International Network for the Demographic Evaluation of Populations and their Health is 45 in 19 low- and middle-income countries where the national and subnational vital registration system generates unreliable population estimates [21, 22]. The nature of data collection of the NN-HDSS is longitudinal These data enable studies of trends in the local area and allow for analyses of social, household, and individual characteristics associated with adolescent pregnancies.

Thus, this study aimed to analyze the trend (2001–2013) in the incidence of adolescent pregnancies in the Cuatro Santos area, northern Nicaragua, based on Health and Demographic Surveillance data and to identify characteristics associated with adolescent pregnancies reported by 20–24-year-old women.

## Methods

### Study setting and population

The Cuatro Santos area, in the northern part of the Chinandega region, Nicaragua, consists of four municipalities of similar population size, with a total of 25,893 inhabitants (2014). This area, 250 km northwest of the capital Managua, is a mountainous terrain bordering Honduras. The climate is predominantly dry, and the traditional source of income has been the cultivation of grains and raising livestock, now with an increasing number of small-scale enterprises. A significant proportion of the population has out-migrated due to economic reasons [23]. In terms of healthcare, the Cuatro Santos area has one larger health center per municipality and the nearest hospital is 130 km distant. The healthcare service has on average five physicians per 10,000 inhabitants. Skilled birth attendance is estimated at 91% and the under-five mortality rate dropped from 40 per 1000 to 20 per 1000 live births between 1990 and 2008 [24–26].

In 1998, local stakeholders in the Cuatro Santos area developed a long-term strategic plan to facilitate multi-dimensional development initiatives to break the cycles of poverty. Interventions included water and sanitation, house construction, microcredits, environmental protection, school breakfasts, technical training, university scholarships, home gardening, breastfeeding promotion, and maternity waiting homes [24]. During the last decade, the proportion of individuals in this region living in poverty was reduced from 79 to 47% [25]. Primary school enrolment increased from 70 to 98%. Under-five mortality dropped from 50 per 1000 live births in 1990 to about 20 per 1000 in 2014 [24–26].

### Northern Nicaragua health and demographic surveillance system (NN-HDSS) and study design

In 2004, a census in the whole Cuatro Santos population covered essential health and demographic information [24]. Surveys followed in 2007, 2009, and 2014 and unique identifiers of households and individuals linked the data. Demographic changes in the households, such as births, deaths, and migration, were registered. Household data included information on the house (floor, walls) and services (water, sanitation, electricity); see Table 1. All women aged 15–49 years living in the households provided retrospective reproductive histories [26]. In the 2009 and 2014 updates, questions covered participation in the following interventions: access to water and latrines, microcredit, home gardening, technical education, school breakfast programs, and telecommunications. Data on food security, household assets, and women’s self-rated health were part of the 2014 update.

**Table 1:** Individual and household variables in the conditional inference tree analyses of adolescent pregnancies NN-HDSS, 2014

**Categorical variables**	**Labels**		**All**^**a**^**20–24** ***n*** **= 1041**		**Stayers**^**a**^**20–24** ***n*** **= 752**	**Leavers**^**a**^**20–24** ***n*** **= 289**		
			**n**	**%**^**b**^	**n**	**%**^**b**^	**n**	**%**^**b**^
**Individual variables**								
Adolescent Pregnancy	0 Not given birth at age 10–19 years		734	71	604	80	130	45
	1 Given birth at age 10–19 years		307	29	148	20	159	55
Occupation	1 Unemployed		65	6	60	8	5	2
	2 Housewife		730	70	462	61	268	93
	3 Employed		64	6	53	7	11	4
	4 Student		182	17	177	24	5	2
Education (years of schooling)	1 No education		131	13	72	10	59	20
	2 Primary (6 yrs)		657	63	455	61	202	70
	3 Secondary (5 yrs)		9	1	9	1	0	0
	4 Higher (5 yrs)		244	23	216	29	28	10
Women’s self-rated health	1 Good		636	61	476	63	160	55
	2 Average or bad		405	39	276	37	129	45
**Household variables**								
UBN^c^	0 No basic need unsatisfied		265	25	210	28	55	19
	1 Wall is made of wood, cartons, plastic AND mud floor		345	33	258	34	87	30
	2 Access to water from rivers, wells, or bought in barrels AND no latrine		421	40	275	37	146	51
	3 Children aged 7 to 14 years are not attending school OR 4 The head is illiterate or not completed primary school AND dependency ratio > 2		10	1	9	1	1	< 1
Poverty	0 Not poor = UBN^c^ 0–1		610	59	468	62	142	49
	1 Poor = UBN^c^ 2–4		431	41	284	38	147	51
House wall type	1 Ceramic brick		302	29	223	30	79	27
	2 Adobe/wattle wall		719	69	524	70	195	67
	3 Other		20	2	5	1	15	5
Water availability	1 Inside pipe		363	35	283	38	80	28
	2 Commune post		32	3	22	3	10	3
	3 Own well		222	21	170	22	52	18
	4 Communal well		292	28	199	26	93	32
	5 River or Creek		76	7	52	7	24	8
	6 Purchased water or Other sources		56	5	26	3	30	10
Toilet type	1 Toilet		23	2	19	3	4	1
	2 Latrine		838	80	658	88	180	62
	3 No toilet or latrine		180	17	75	10	105	36
Floor in house	1 Ceramic brick		85	8	68	9	17	6
	2 Brick of Mud or Cement		73	7	63	8	10	3
	3 Tiling		331	32	248	33	83	29
	4 Mud floor		552	53	373	50	179	62
Electricity in the house	1 Yes		931	89	698	93	233	81
	2 No		110	11	54	7	56	19
Stove in house	1 Gas		81	8	52	7	29	10
	2 Wood		960	92	700	93	260	90
Water meter in use	1 Yes		216	21	167	22	49	17
	2 No		825	79	585	78	240	83
Microcredit in HH^c^	1 Yes		138	13	108	14	30	10
	2 No		903	87	644	86	259	90
Technical training in HH^d^	1 Yes		162	16	133	18	29	10
	2 No		879	84	619	82	260	90
Home garden in HH^d^	1 Yes		61	6	49	7	12	4
	2 No		980	94	703	93	277	96
Home garden in use	1 Yes		43	4	35	5	8	3
	2 No		998	96	717	95	281	97
Anxiety in HH^d^ for lack of food	0 Never		166	16	122	16	44	15
	1 Rarely (1–2 times)		429	41	299	40	130	45
	2 Sometimes (3–10 times)		241	23	175	23	66	23
	3 Often (> 10 times)		205	20	156	21	49	17
Inability in HH^d^ to eat preferred food	0 Never		163	16	111	15	52	18
	1 Rarely (1–2 times)		436	42	307	41	129	45
	2 Sometimes (3–10 times)		344	33	260	35	84	29
	3 Often (> 10 times)		98	9	74	10	24	8
Limited variation of food in HH^d^ due to lack of food	0 Never		221	21	157	21	64	22
	1 Rarely (1–2 times)		483	46	344	46	139	48
	2 Sometimes (3–10 times)		267	26	197	26	70	24
	3 Often (> 10 times)		70	7	54	7	16	6
Few kinds of food consumed in HH^d^ due to lack of food	0 Never		212	20	154	7	58	20
	1 Rarely (1–2 times)		505	49	358	48	147	51
	2 Sometimes (3–10 times)		264	25	192	26	72	25
	3 Often (> 10 times)		60	6	48	6	12	4
Reduction of portion sizes of meals in HH^d^ due to lack of food	0 Never		295	28	213	28	82	28
	1 Rarely (1–2 times)		494	47	348	46	146	51
	2 Sometimes (3–10 times)		207	20	155	21	52	18
	3 Often (> 10 times)		45	4	36	5	9	3
Fewer meals consumed in HH^d^ due to lack of food	0 Never		454	44	331	44	123	43
	1 Rarely (1–2 times)		408	39	285	38	123	43
	2 Sometimes (3–10 times)		149	14	112	15	37	13
	3 Often (> 10 times)		30	3	24	3	6	2
No food to eat in HH^d^ due to lack of resources	0 Never		757	73	546	73	211	73
	1 Rarely (1–2 times)		231	22	164	22	67	23
	2 Sometimes (3–10 times) and often (> 10 times)		53	5	42	6	11	4
HH^d^ going to sleep hungry due to lack of food	0 Never		913	88	654	87	259	90
	1 Rarely (1–2 times)		97	9	72	10	25	9
	2 Sometimes (3–10 times) and often (> 10 times)		31	3	26	3	5	2
HH^d^ having days of hunger due to insufficient amount of food	0 Never		958	92	688	91	270	93
	1 Rarely (1–2 times)		62	6	45	6	17	6
	2 Sometimes (3–10 times) and often (> 10 times)		21	2	19	3	2	1
TV antenna in HH^b^	1 Parabolic antenna		149	14	125	17	24	8
	2 Normal antenna		427	41	334	44	93	32
	3 Handmade antenna		88	8	72	10	16	6
	4 No antenna		377	36	221	29	156	54
Car in HH^d^	1 Yes		31	3	28	4	3	1
	2 No		1010	97	724	96	286	99
Motorbike in HH^d^	1Yes		123	12	88	12	35	12
	2 No		918	88	664	88	254	88
Bike in HH^d^	1 Yes		184	18	133	18	51	18
	2 No		857	82	619	82	238	82
Horse in HH^d^	1 Yes		296	28	241	32	55	19
	2 No		745	72	511	68	234	81
Refrigerator in HH^d^	1 Yes		363	35	298	40	65	22
	2 No		678	65	454	60	224	78
Sewing machine in HH^d^	1 Yes		69	7	62	8	7	2
	2 No		972	93	690	92	282	98
Computer in HH^d^	1 Yes		56	5	44	6	12	4
	2 No		985	95	708	94	277	96
Tortilla oven in HH^d^	1 Yes		192	18	173	23	19	7
	2 No		849	82	579	77	270	93
Stove with a chimney in HH^d^	1 Yes		21	2	15	2	6	2
	2 No		1020	98	737	98	283	98
Sex of HH^d^ head	1 Female head of HH^d^		250	24	208	28	42	15
	2 Male head of HH^d^		791	76	544	72	247	85
Illiterate living in HH^d^	0 No illiterate in HH^d^		749	72	508	68	241	83
	1 Illiterate in HH^d^		292	28	244	32	48	17
Highest education in HH^d^	0 No education or Primary school		174	17	82	11	92	32
	2 Secondary school		522	50	374	50	148	51
	3 Technical education		82	8	67	9	15	5
	4 University education		263	25	229	30	34	12
Immigration in HH^d^	0 No immigration in HH^d^		464	45	412	55	52	18
	1 Immigration in HH^d^		577	55	340	45	237	82
Emigration in HH^d^	0 No emigration in HH^d^		382	37	207	28	175	61
	1 Emigration in HH^d^		659	63	545	72	114	39
HH^c^ member immigrated from a foreign country	0 No immigration from another country in a household		938	90	658	88	280	97
	1 Immigration from another country in HH^d^		103	10	94	12	9	3
HH^d^ member emigrated to a foreign country	0 No emigration to another country in HH^d^		880	85	621	83	259	90
	1 Emigration to another country in HH^d^		161	15	131	17	30	10
Children (< 15 yrs.) In HH^d^ working	0 No		1017	98	732	97	285	99
	1 Yes		24	2	20	3	4	1
**Continuous variables**								
	**All**^**b**^**20–24** ***n*** **= 1041**		**Stayers**^**b**^**20–24** ***n*** **= 752**			**Leavers**^**b**^**20–24** ***n*** **= 289**		
	Mean (Median)	Min/ Max	Mean (Median)		Min/ Max	Mean (Median)	Min/ Max	
No of adults in HH^d^	6.2 (6.0)	1/19	7.0 (7.0)		2/19	4.2 (2.0)	1/17	
No in HH^d^ not working	3.4 (3.0)	0/13	4.0 (4.0)		0/13	1.9 (1.0)	0/9	
No in HH^d^ working	1.7 (1.0)	0/6	1.9 (2.0)		0/6	1.3 (1.0)	0/5	
No of working adults (> = 15 yrs.) in HH^d^	1.7 (1.0)	0/6	1.9 (2.0)		0/6	1.3 (1.0)	0/5	
No of not working adults (> = 15 yrs.) in HH^d^	3.4 (3.0)	0/13	3.0 (3.0)		0/8	1.4 (1.0)	0/6	
No of individuals in HH^d^	8.1 (8.0)	1/25	8.9 (8.0)		2/25	5.9 (4.0)	1/20	
Ratio of adults working to not working in HH^d^	0.9 (0.8)	0/9	0.8 (0.7)		0/9	1.2 (1.0)	0/9	
Ratio of working adults (> = 15 yrs.) to no of individuals in HH^d^	0.2 (0.2)	0/1	0.2 (0.2)		0/0.8	0.26 (0.25)	0/1	
Proportion of adolescent pregnancies in the home community 2014	0.3 (0.3)	0/1	0.3 (0.3)		0/0.8	0.4 (0.3)	0/1	

^a^All are all women that have reported about pregnancies in each age category. *Stayers* are those we presume have stayed in the household they belonged to when getting pregnant (or at an earlier age), due to that they either were daughters or had another family relation to the head of household. *Leavers* are those we presume have left the household they belonged to before getting pregnant (or at an earlier age), due to that they were head of household or spouse to head of household, were not family of the head of household, or employees

^b^Due to rounding, percentages do not always add up to 100

^c^*UBN* Unsatisfied Basic Need index

^d^*HH* Households

Trained local women with at least high school education conducted the fieldwork with careful supervision. Forms were checked before computerization and returned to the field if the information was missing or suspected to be incorrect. Further quality controls after computerization included logical checks of data. Researchers carefully cleaned the data and stored these in relational databases.

### Outcome variable

The outcome variable for incidence calculations and Conditional Inference Trees (CIT) analyses, adolescent pregnancy (yes/no), was derived by taking the first pregnancy in women 20–24 years of age and the result of that pregnancy (live birth, stillbirth, abortion) into account. The same outcome covered different age categories and cohorts, showing trends in ABR and APR, respectively. The ABR is defined as live births per 1000 women 10–14 years old and 15–19 years old, and the APR as live births, ongoing pregnancies, abortions, and stillbirths per 1000 women in the same age categories.

### Predictor variables

The predictor variables on the individual level included in the CIT analyses were merged with variables at the household level referred to each individual using housing ID, for variable list see Table 1. We included occupation (unemployed, housewife, employed, student) and education (no education, primary, secondary, higher) as reported by each woman. Also, women’s self-rated health was assessed at the time of the interview by a five-point Likert scale based on the following question: “In general, how would you assess your health today?” The interviewer provided the following options: very good, good, medium, bad, or very bad. In the analyses this information was classified as good (very good, good, medium) and bad (bad, very bad) health, respectively.

The household was defined as persons residing in the household at that time. The Unsatisfied Basic Needs index [27] was composed of four components: (1) housing conditions (unsatisfied: walls of wood, cardboard, plastic and earthen floor); (2) access to water and latrine (unsatisfied: water from river, well, or bought in barrels and no latrine or toilet); (3) school enrolment of children (unsatisfied: any children 7–14 years of age not attending school); and (4) education of head of the family and ratio of dependent (< 15 yrs. and > 65 yrs.) household members to working-age members (15–65 yrs.) (unsatisfied: head of the family illiterate or dropped out of primary school and ratio of dependent household members to working-age members. > 2.0). Each component rendered a score of zero if satisfied, and one, if unsatisfied. Thus, the total sum varied from zero to four. Households with zero or one unsatisfied basic need were considered non-poor, while poor households had two to four unsatisfied basic needs [25]. Characteristics of houses and households were also included in the analyses, such as the material of walls, floor, access to electricity, type of stove, access to water, and type of toilet. The interventions implemented in the area were represented by household-related information on such participation. The presence of a water meter indicated that the household had got water installed as part of the last decade’s interventions. Also, information was included on previous and current participation in home gardening, if anyone in the household had received microcredit or had participated in technical training.

The nine-item Household Food Insecurity Access Scale, version 3, was used [28]. This scale covers experiences regarding 1) anxiety in the household due to lack of food; 2) inability to eat preferred food because of lack of resources; 3) limited variety of food due to lack of resources; 4) consumption of few kinds of food because of lack of resources; 5) reduction of portion sizes of meals due to lack of food; 6) consumption of fewer meals per day because of lack of food; 7) no food to eat in the household because lack of resources; 8) going to sleep at night hungry due to lack of food, and 9) days of hunger because of insufficient amounts of food to eat. The respondents were either the head of the household or the person responsible for the household expenditure and food preparation and they reported on the food security situation during the last 4 weeks. For each affirmative answer, the person provided additional information on the frequency in a four-point scale (never, rarely, sometimes, often).

Included household assets were having a TV antenna, car, motorbike, bike, horse, refrigerator, sewing machine, computer, tortilla oven, and a chimney for the wood-burning stove.

We also included gender of household head, any illiteracy, the highest education level in the household (none, primary, secondary, technical, university education) and if the household had children below age 15, working. Migration was defined as a household member aged 18–65 who migrated in or out of the household since the latest update (5 yrs.) and data were included on the household level on in- and out-migration, including to and from foreign countries.

We constructed variables on the number of adults and children living in the household, number of adults and children working in the household, number of adults not working in the household, and the ratio between adults working and not working in household, as well as the ratio between adults working and number of individuals in the household (see Table 1). We also included a variable on the community level adolescent pregnancy proportion. A community in Cuatro Santos is a group of households with geographical proximity, and for the 2014 cycle, we counted 71 communities with a mean of 81.6 of households (SD 58.01) in each community. The adolescent pregnancy proportion was calculated as the percentage of pregnancies in 10–19-year-old females per community as reported at the moment of the 2014 interview by women aged 20–24 that gave the first birth between 10 and 19 years of age. In total, the data set contained 53 variables.

### Analytical methods

For the annual rate of ABR (live births per 1000 women 10–14 and 15–19 years of age, respectively) and APR (live births, ongoing pregnancies, abortions, and stillbirths per 1000 women in the same age groups) we used the first live birth at 10–14 and 15–19 years of age. We included reports by women aged 15–19 and 20–24 at the time of the interview.

**Fig. 1:**
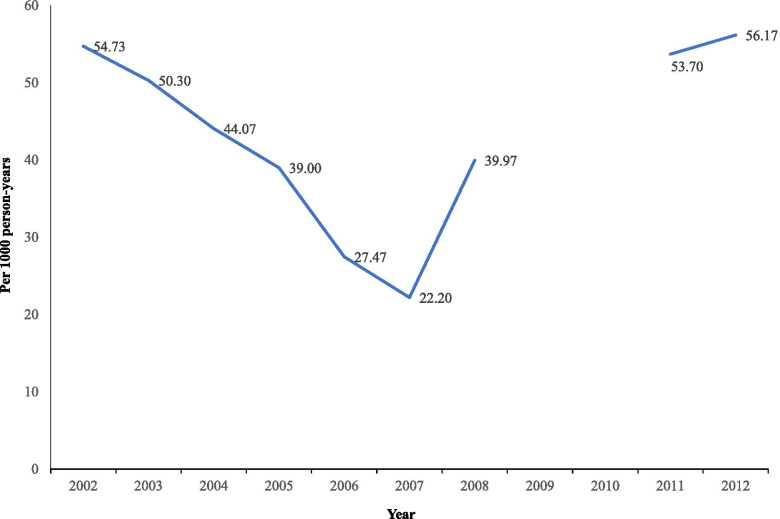
Three years moving averages of incidence rates of pregnancy in adolescents (15-19 years of age), Cuatro Santos, Nicaragua, 2001–2013

We determined the annual incidence rate of pregnancies between 15 and 19 years (per 1000 person-years) for the 3 years preceding the survey using the first birth reported by women aged 20–24, at the time of interview for each NN-HDSS cycle. We calculated three-years moving averages of incidence rates to display the incidence trend (Fig. 1). We based the 2006 rate on averaged data from the 2007 and 2009 cycles. The time between the two last cycles was 5 years, which implies that there were no calculated incidences for 2009 and 2010. We used the Cohort software (Department of Epidemiology and Global Health in cooperation with Umeå University data center, Umeå, Sweden) to calculate person time in the study.

**Fig. 2:**
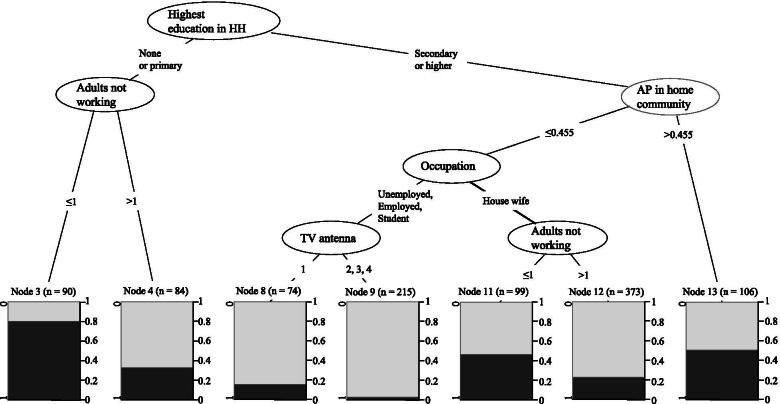
Cross-validated conditional inference tree, where each end node includes at least 50 individuals. Black areas in end nodes show proportions of women 20-24-years-old who experienced adolescent pregnancies (incl. ongoing pregnancies, stillbirths, and abortions) and grey areas women 20-24-years-old who have not experienced any adolescent pregnancy. The unit of analysis is the individual, but individual variables included were merged with variables at the household and community level referred to each individual using housing ID. AP = Adolescent pregnancy

The CIT analyses included all women in the 20–24 age group with the outcome of adolescent pregnancy (yes/no) and in subsets of data on stayers and *leavers* as presented below. The number of candidate predictors evaluated for inclusion was 52 (Table 1, Fig. 2, Additional file 1: Fig. S1 and Additional file 2: Fig. S2). CIT is one of the newer decision tree frameworks used in data mining that allows for specifying an arbitrarily high number of predictor variables, handling variables of different types, automatically discovering complex interactions between predictor variables, and including them into the model [29, 30]. The method embeds a statistical hypothesis-testing framework into a recursive partitioning algorithm for model building [30].

The informants relatively often reported individual and household-level information used as predictors after having an adolescent pregnancy. Thus, these variables may be a consequence of the outcome (adolescent pregnancy) rather than a ‘risk factor’ for the outcome. To restrict the possibility of this error, we split the data into two subsets labeled “*stayers*” and “*leavers*.” These two subsets of data were analyzed separately for 20–24-year-old women. *Stayers*, we presumed, had stayed in the household they belonged to at the time of pregnancy (or at an earlier age). They were either daughters or had another family relation to the head of the household rather than being the partner. *Leavers* were those presumed to have left the home they were associated with before getting pregnant (or at an earlier age), based on that they were head of household or spouse to head of household, i.e., they were not family to the head of household or employees. Thus, by using these two subsets, the household variables should be similar for *stayers* as when they got pregnant but different and maybe a consequence of the adolescent pregnancy, for the *leavers*.

Cross-validation, a well-established method, was applied to select the tree of optimal size and the best predictive performance [31]. The minimum number of observations in each terminal node (subgroup) was limited to 50 to ensure public health significance. We used programming language R version 3.2.4 [32] and the “party” package [33] for all analyses.

## Results

In the 2014 Northern Nicaraguan HDSS update, 5233 households were inhabited and provided data. The total number of 15–19-year-old and 20–24-year-old women included in the calculation of ABR and APR in the four cycles of the NN-HDSS varied between 865 and 1623 (Table 2). See Table 3 for the total number of women aged 10–19 years with pregnancies and the person-years included in the incidence calculations of adolescent pregnancies. The CIT analysis included data on 1041 20–24-year-old women after excluding individuals with missing values. Table 1 shows the characteristics of the included women.

**Table 2:** Adolescent birth rates and pregnancy rates reported among 15–19-years-olds and 20–24-years-olds by cycle of NN-HDSS, 2004–2014

NN-HDSS cycle	2004	2007	2009	2014
**Age group 15–19**				
No. of women	1273	1467	1623	1389
Live Births (10–19)	146	150	126	186
Adolescent Birth Rate ABR (95%CI)	114.7 (97.1–132)	102.2 (86.7–117.8)	77.6 (64.6–90.6)	133.9 (116.0–151.8)
Live births (10–14)	14	13	5	18
Adolescent Birth Rate ABR (95%CI)	11.0 (5.2–16.7)	8.9 (4.0–13.6)	3.1 (0.3–5.7)	13.0 (7.0–18.9)
Live births (15–19)	132	137	121	168
Adolescent Birth Rate ABR (95%CI)	103.7 (86.9–120.4)	93.4 (78.5–108.3)	74.6 (61.7–87.3)	121.0 (103.8–138-1)
No. of women	1273	1467	1623	1389
Pregnancies 10–19	201	187	148	241
Adolescent Pregnancy Rates APR (95%CI)	157.9 (137.9–177.9)	127.5 (110.4–144.5)	91.2 (77.1–105.2)	173.5 (153.6–193.4)
Pregnancies 10–14	16	14	6	18
Adolescent Pregnancy Rates APR (95%CI)	12.6 (6.4–18.6)	9.5 (4.5–14.5)	3.7 (0.7–6.6)	13.0 (7.0–18.9)
Pregnancies 15–19	185	173	142	223
Adolescent Pregnancy Rates APR (95%CI)	145.3 (126.0–164.7)	117.9 (101.4–134.4)	87.5 (73.7–101.2)	160.5 (141.2–179.9)
**Age group 20**–**24**				
No. of women	982	865	886	1292
Live Births (10–19)	406	188	118	423
Adolescent Birth Rate ABR (95%CI)	413.4 (382.6–444.2)	217.3 (189.9–244.8)	133.2 (110.8–155.6)	327.4 (301.8–353.0)
Live births (10–14)	24	8	7	13
Adolescent Birth Rate ABR (95%CI)	24.4 (14.7–34.1)	9.2 (2.8–15.6)	7.9 (2.0–13.7)	10.1 (4.6–15.5)
Live births (15–19)	382	180	111	410
Adolescent Birth Rate ABR (95%CI)	389.0 (358.5–419.5)	208.1 (181.0–235.1)	125.3 (103.5–147.1)	317.3 (292.0–342.7)
No. of women	982	865	886	1292
Pregnancies 10–19	427	193	123	447
Adolescent Pregnancy Rates APR (95%CI)	434.8 (403.8–465.8)	223.1 (195.4–250.9)	138.8 (116.1–161.6)	346.0 (320.0–371.9)
Pregnancies 10–14	26	8	7	14
Adolescent Pregnancy Rates APR (95%CI)	26.4 (16.4–36.5)	9.2 (2.8–15.6)	7.9 (2.0–13.7)	10.8 (5.1–16.4)
Pregnancies 15–19	401	185	116	433
Adolescent Pregnancy Rates APR (95%CI)	408.3 (377.6–439.1)	213.9 (186.5–241.2)	130.9 (108.7–153.1)	335.1 (309.4–360.9)

**Table 3:** Incidence rates of pregnancies per person-years in women aged 15–19 years in the NN-HDSS cycles as reported by 20–24-years-old women. The rates were calculated for the 3 years preceding the survey

Year	Pregnancies (15–19-years)	Mean age at pregnancy (years of age)	Person-years	Crude incidence × 1000 person-years
Baseline (end 2003/2004)				
2001	43	18.7	572.4	75.1
2002	27	19.2	503.6	53.6
2003	17	19.6	440.4	38.5
First cycle (2007)				
2004	45	18.2	728.0	61.8
2005	23	19.0	657.2	34.9
2006	14	19.4	603.4	23.2
Second cycle (2009)				
2006	14	18.6	799.6	17.5
2007	21	18.7	770.0	27.2
2008	14	19.4	729.6	19.1
Third cycle (2014)				
2011	68	18.4	923.3	73.6
2012	56	19.0	817.9	68.4
2013	19	19.3	716.3	26.5

### Trends of adolescent births and pregnancies 2004–14 in Cuatro Santos, Nicaragua

Table 2 provides the ABR and APR for girls and young women 10–14 years of age and 15–19-years of age. Overall, both ABR and APR decreased from 2004 to 2009, followed by an increase in 2014. The difference between reported live births and pregnancies was substantial, especially in the younger age group. In the age group 15–19 years, 71–85% were live births, and 15–29% constituted present pregnancies, stillbirths, or abortions. In the older age group, the proportion of stillbirths and abortions was 3–5% of all pregnancies. In the 10–14 years group, 0–7% of pregnancies were stillbirths or abortions, as reported by both age groups of informants.

### Incidence trend of adolescent pregnancies 2001–2013 in Cuatro Santos, Nicaragua

The incidence rates of pregnancies per 1000 person-years for women 15–19 years of age for the cycles of the NN-HDSS varied from 17.5 to 75.1, as seen in Table 3. The trend analysis (Fig. 1) showed a steep decline in the incidence of adolescent pregnancies from 2001 to 2007, followed by a steep upwards turn to 2008, and after that, an increase to higher levels 2011–2012.

### Predictors for adolescent pregnancies reported by 20–24-year-old women

In the CIT analysis, including all 20–24-year-old women (*n* = 1041), the most crucial splitting variable was “highest education level in the household,” followed by “non-working adults in the household” and “proportion of adolescent pregnancies in the community” (Fig. 2). Figure 2, (node eight and nine, *n* = 74 + 215) shows the subgroups of women with the least likelihood of having experienced a pregnancy in adolescence. They were those who lived in a household with secondary or higher education, in a community with a lower level of adolescent pregnancies (≤ 0.455, the mean was 0.3 for this variable and group of women as seen in Table 1), and were not housewives. Women with the highest likelihood of having experienced an adolescent pregnancy (Fig. 2, node three, *n* = 90) lived in a household with no education or only primary school, and where the number of adults not working was one or zero. The second highest likelihood of having experienced an adolescent pregnancy (Fig. 2, node 13, *n* = 106) had women who lived in a household with secondary school or higher and in a community, where the proportion of adolescent pregnancies was higher (> 0.455, the mean was 0.3 for this variable and group of women as seen in Table 1).

The analysis of 20–24-year-old *stayers* (presumed to have stayed in the household they belonged to when getting pregnant, or at an earlier age, *n* = 752, Additional file, Fig. S1) showed that women with a higher proportion of adolescent pregnancy in the community and with no education or primary school showed the highest occurrence of adolescent pregnancies.

Additional file, Fig. S2 shows the 20–24-year-old *leavers* (presumed to have left the household they belonged to before getting pregnant, or at an earlier age). Among *leavers*, the highest proportion of pregnancies was found in the group with no education, followed by those with primary or higher education and a higher percentage of adolescent pregnancies in the community.

## Discussion

To our knowledge, this is the first study that examined recent time-trend data of adolescent pregnancy from rural settings through a valid prospective demographic surveillance system and analyzed a large number of related factors that classical statistical methods are unable to handle. In these Northern Nicaraguan communities, adolescent pregnancies and live births decreased from 2004 to 2009, followed by a marked increase up to 2014. The adolescent pregnancy incidence rates 2001-2013 had a similar shape. The curve steadily dropped from 2001 to 2007, followed by a steep upward trend from 2007 to 2008 and increasing even more during the two last years of study. The 20-24-year-old women, who had experienced an adolescent pregnancy, more frequently lived in a household with a low education level and where most adults were working. Further, the proportion of adolescent pregnancies in the home community was positively associated with a higher occurrence of adolescent pregnancies. Our results are generalizable to the rural areas of Nicaragua and similar settings in Central America and the Caribbean, however the specific findings related to the context might vary from setting to setting.

Almost all literature on ‘risk factors’ for adolescent pregnancies refers to results on births retrospectively reported by teenage mothers, studied by cross-sectional designs. As this approach does not capture the temporality of risk factors, it implies that many reported risk factors might be the consequences of adolescent pregnancy, for example, marriage, low education, and low income. Neal and co-authors also suggested this in 2018 [12]. A more appropriate labeling would be to describe the identified factors as associated with the retrospectively reported adolescent birth.

We tried to overcome the temporality problem by splitting our data set into *stayers* and *leavers*; however, that action only partly solved the problem, since individual variables in most cases were collected after the pregnancy. Nevertheless, as the household variables could be the same among the *stayers* as when the pregnancy happened, while they probably have changed for the *leavers*, it can explain the difference seen in the CIT analysis on the community adolescent pregnancy proportion being more critical among the *stayers* than among the *leavers*.

The decrease 2004-2007 of ABR for the 15-19-year group coincided with the country decline reported in PAHO-2019 (2004-7), e.g., the overall ABR changing from 111.5 to 106.4 [20]. A study that examined data from four nationally representative surveys from 1987 to 2007 in Central America showed that the percentage of adolescents, who had had a live birth in Nicaragua, was the highest, 26% in 1987, but after that reduced to 20% in 2007 [34].

The strong association between a low educational level and adolescent pregnancy is probably, at least partly, a consequence of adolescent pregnancy, forcing pregnant teenagers to leave school. This contrasts with the law that prohibits public schools from expelling girls who become pregnant (Nicaraguan Child and Adolescence Code Law, Law No. 287). Irrespective of the law, social pressure makes girls leave school. Our results indicate that women in their 20ies, who had an adolescent pregnancy, were not able to overcome this educational disadvantage.

Living in households with many working adults was common among women who had experienced an adolescent pregnancy. This fact contradicts earlier reported associations with lower wealth [11, 15, 16]. Similarly, no variable measuring wealth or poverty showed to be associated with adolescent pregnancy. However, few present adults might point to inadequate supervision of adolescents that may increase the risk of pregnancy.

The occurrence of adolescent pregnancies in the local community as a significant factor points to the influence of contextual values in the community on teenage pregnancies. A similar result was reported from an analysis of the latest Nicaraguan DHS data, where a high proportion of women having a child increased the occurrence of teen births [35].

A study using the 2001 Nicaraguan Demographic and Health Surveillance data concluded that age at sexual debut was the most influential risk factor and that lack of health care contributed to adolescent pregnancies [36]. That report described the Nicaraguan culture surrounding sex and childbearing as influenced by machismo and marital instability, where Nicaraguan men sought to prove their masculinity by fathering numerous children. Despite this, young women tried to cement their union by having a child. This culture was reportedly the background to the persistently high rate of adolescent pregnancies in the country [36]. A recent study from a context similar to the Cuatro Santos area showed that young girls had less knowledge of sexual and reproductive health, compared to young men and older adolescents [37].

We found an increasing trend in teenage pregnancies over 2009-2014 in our study population. Despite our trend results were not in line to national figures [20], in other LMIC, increasing trends have been experienced in underserved population groups [38–40]. Therefore, our findings support the interest in monitoring adolescent pregnancy in disaggregated subgroups (e.g., geographic and social stratifiers) within the country since subnational-specific health risks seem to vary from in-country targets [41].

The health and demographic surveillance data have shown to be of high quality [24, 25], and cover the whole population in the Cuatro Santos area with very few non-participants. Data on pregnancies in the 10-14-years group are not reliable since the questions in the NN-HDSS questionnaires focused on pregnancies from 15 years of age. Surveys on birth and pregnancy history might be subject to recall bias. To address this bias, we analyzed data from the survey in the three preceding years which is a time used in similar surveys with good quality fertility estimations in low- and middle- income countries [42]. Furthermore, we do not have data on proximal predictors, such as access to reproductive health services, including effective contraception and activities related to sexual violence, gender norms, or status of motherhood as a cultural value. Finally, the CI decision-tree enabled us to simultaneously include and assess the importance of a relatively large set of predictor variables with the outcome of adolescent pregnancy. This method also automatically includes and evaluates interactions between the predictors. The output from a CI tree analysis displays precise information about the direction, size, and priority order of the found associations.

## Conclusion

A high incidence of adolescent pregnancies was present in the Cuatro Santos area. There was a steep decline from 2001 to 2007 that was reversed the following years up to 2014. Low education, a high number of working adults in the household, and a high proportion of adolescent pregnancies in the home community were associated with adolescent pregnancies. Household assets reflecting wealth, poverty, or participating in interventions were not linked to teenage pregnancies.

The importance of the level of adolescent pregnancies in the local community indicate that solutions also need to be sought in the context influencing the culture of early motherhood.

## Supplementary Information

**Additional file 1: Figure S1.** Cross-validated conditional inference tree, where each end node includes at least 50 individuals. Black areas in end nodes show proportions of 20-24-years-old women stayers (presumed to have stayed in the household they belonged to when getting pregnant or at an earlier age) who experienced adolescent pregnancies (incl. ongoing pregnancies, stillbirths, and abortions) and grey areas women 20-24-years-old that have not experienced any adolescent pregnancy. The unit of analysis is the individual, but individual variables included were merged with variables at the household and community level referred to each individual using housing ID. AP = Adolescent pregnancy.

**Additional file 2: Figure S2.** Cross-validated conditional inference tree, where each end node includes at least 50 individuals. Black areas in end nodes show proportions of 20-24-year-old women classified as leavers who experienced adolescent pregnancies (incl. ongoing pregnancies, stillbirths, and abortions) and grey areas women 20-24-year-old classified as leavers that have not experienced any adolescent pregnancy. The unit of analysis is the individual, but individual variables included were merged with variables at the household and community level referred to each individual using housing ID. AP = Adolescent pregnancy.

## Data Availability

The raw data supporting the conclusions of this manuscript will be made available without undue reservation, to any qualified researcher from the corresponding author on reasonable request (wperezc2018@gmail.com).

## Abbreviations

ABR: Adolescent birth rate
APR: Adolescent pregnancy rates
GDP: Gross domestic product
CIT: Conditional Inference Trees
NN-HDSS: Northern Nicaragua Health and Demographic Surveillance System
SD: Standard Deviation
SDG: Sustainable Development Goals
UBN: Unsatisfied Basic Need Index
