# Supplementary material for: Trends and factors related to adolescent pregnancies: an incidence trend and conditional inference trees analysis of northern Nicaragua demographic surveillance data
Source: BMC Pregnancy Childbirth. 2021 Nov 5;21:749. doi: 10.1186/s12884-021-04215-4 (PMC8569964; doi:10.1186/s12884-021-04215-4)
Supplement: Supplementary file 1 — Additional file 1: Figure S1. Cross-validated conditional inference tree, where each end node includes at least 50 individuals. Black areas in end nodes show proportions of 20-24-years-old women stayers (presumed to have stayed in the household they belonged to when getting pregnant or at an earlier age) who experienced adolescent pregnancies (incl. ongoing pregnancies, stillbirths, and abortions) and grey areas women 20-24-years-old that have not experienced any adolescent pregnancy. The unit of analysis is the individual, but individual variables included were merged with variables at the household and community level referred to each individual using housing ID. AP = Adolescent pregnancy. [file 12884_2021_4215_MOESM1_ESM.docx]

Additional file 1 Figure S1

Cross-validated conditional inference tree, where each end node includes at least 50 individuals. Black areas in end nodes show proportions of 20-24-years-old women stayers (presumed to have stayed in the household they belonged to when getting pregnant or at an earlier age) who experienced adolescent pregnancies (incl. ongoing pregnancies, stillbirths, and abortions) and grey areas women 20-24-years-old that have not experienced any adolescent pregnancy. The unit of analysis is the individual, but individual variables included were merged with variables at the household and community level referred to each individual using housing ID. AP=Adolescent pregnancy.
